# Beyond Inflammation: Associations of Depression and Anxiety With Fatigue in Rheumatoid Arthritis

**DOI:** 10.7759/cureus.109695

**Published:** 2026-05-26

**Authors:** Nadina Kurtanović, Maida Skokić, Melika Salihović Jahić, Nera Hodžić, Ena Gogić, Amra Tulumović Mešanović, Alma Šerak Tokić, Mirza Babić

**Affiliations:** 1 Department of Physical Medicine and Rehabilitation, Health Institution Spa Gata Bihać, Bihać, BIH; 2 Department of Rheumatology, University Clinical Center Tuzla, Tuzla, BIH; 3 Department of Psychiatry, University Clinical Center Tuzla, Tuzla, BIH; 4 Clinic for Physical Medicine and Rehabilitation, Clinical Center University of Sarajevo, Sarajevo, BIH; 5 Department of Internal Medicine, Cantonal Hospital Bihać, Bihać, BIH

**Keywords:** anxiety, biopsychosocial model, depression, disease activity, fatique, rheumathoid arthritis

## Abstract

Background

Fatigue is one of the most common and disabling symptoms in patients with rheumatoid arthritis (RA). Although often attributed to inflammatory disease activity, psychological factors such as depression and anxiety may also contribute significantly. This study aimed to evaluate the association between fatigue and depressive and anxiety symptoms in patients with RA and to determine whether these associations persist after adjustment for clinical and demographic variables.

Methods

In this cross-sectional secondary analysis, 90 patients with rheumatoid arthritis were included. Fatigue was assessed using the Functional Assessment of Chronic Illness Therapy-Fatigue (FACIT-F) questionnaire. Depressive and anxiety symptoms were measured using the Beck Depression Inventory-II (BDI-II) and the Beck Anxiety Inventory (BAI). Disease activity was evaluated using the Disease Activity Score in 28 joints (DAS28). Associations were analyzed using Spearman’s correlation, and multivariable linear regression models were used to identify independent predictors of fatigue.

Results

The median FACIT-F score was 37.0 (interquartile range (IQR): 27.0-45.0), although moderate-to-severe fatigue was present in 32 (35.6%) patients. Fatigue was moderately associated with disease activity (ρ = -0.38, p < 0.001) and strongly associated with depressive (ρ = -0.63, p < 0.001) and anxiety symptoms (ρ = -0.60, p < 0.001). In multivariable analysis, both depressive (β = -0.50, p = 0.008) and anxiety symptoms (β = -0.40, p = 0.002) remained independently associated with fatigue. Inclusion of psychological variables increased the explained variance in fatigue from 19.8% to 52.4% (ΔR² = 0.326).

Conclusion

Fatigue in rheumatoid arthritis appears to be associated not only with inflammatory disease activity but also with depressive and anxiety symptoms. These findings support a multidimensional biopsychosocial understanding of fatigue and indicate that psychological factors are important correlates of fatigue severity in rheumatoid arthritis.

## Introduction

Fatigue is one of the most prevalent and disabling symptoms in rheumatoid arthritis (RA), affecting up to 70% of patients and significantly impairing quality of life [1,2]. It is frequently reported to be as difficult to manage as pain [3] and has a substantial impact on physical functioning, social participation, and overall well-being. Although fatigue is often attributed to inflammatory disease activity, growing evidence suggests that its etiology is multifactorial and extends beyond purely biological mechanisms [4,5].

Psychological symptoms, particularly depression and anxiety, are common comorbidities in RA and have been associated with both disease activity and functional outcomes [6,7]. Even in early disease stages, depressive and anxiety symptoms have been linked to higher disease activity and worse patient-reported outcomes [8]. In addition, previous studies have reported associations between psychological distress and fatigue across chronic inflammatory conditions [9,10]. However, the relative contribution of inflammatory and psychological factors to fatigue remains incompletely understood, reflecting the complex interplay of multiple contributing mechanisms [11]. Disease activity indices such as the Disease Activity Score in 28 joints (DAS28) are widely used to assess inflammatory burden in RA, yet they do not fully explain variability in fatigue severity [4]. This has contributed to the development of biopsychosocial models, in which fatigue is conceptualized as the result of interactions between biological, psychological, and social factors [10].

Although previous studies have examined fatigue in relation to psychological factors and disease activity in rheumatoid arthritis, their combined and relative contributions within a single analytical framework remain insufficiently explored. The present study addresses this gap by integrating psychological and clinical variables within a single cohort and quantifying their relative contributions to fatigue severity. Understanding the relative contribution of depressive and anxiety symptoms to fatigue may be clinically relevant, particularly in patients reporting persistent fatigue despite treatment of inflammatory disease activity. If psychological distress contributes substantially to fatigue beyond inflammatory activity, comprehensive management approaches targeting mental health may be justified alongside pharmacologic control of RA.

This cross-sectional secondary analysis aimed to evaluate fatigue severity, measured using the Functional Assessment of Chronic Illness Therapy-Fatigue (FACIT-F) questionnaire, in patients with rheumatoid arthritis and to examine whether depressive and anxiety symptoms were independently associated with fatigue after adjustment for disease activity and relevant clinical and demographic variables.

## Materials and methods

This cross-sectional secondary analysis was conducted using data collected between November 2025 and March 2026 under two previously approved study protocols investigating clinical and patient-reported outcomes in rheumatoid arthritis. The first protocol evaluated depressive and anxiety symptoms, while the second assessed fatigue. Both protocols were conducted within the same cohort of patients attending routine follow-up visits at the Department of Rheumatology at the University Clinical Center Tuzla. In the present analysis, fatigue severity assessed using FACIT-F was predefined as the primary outcome variable, while depressive and anxiety symptoms (Beck Depression Inventory-II (BDI-II) and Beck Anxiety Inventory (BAI) scores) were analyzed as the principal explanatory variables.

A total of 100 adult patients (≥18 years) with rheumatoid arthritis, diagnosed according to the 2010 American College of Rheumatology (ACR)/European League Against Rheumatism (EULAR) classification criteria, were consecutively recruited during routine outpatient visits, meaning that all eligible patients attending during the study period who met the predefined eligibility criteria were invited to participate. Patients presenting with acute infection, active malignancy, or severe anemia were excluded at enrollment. After applying these exclusion criteria, the final analytical sample comprised 90 patients.

Fatigue assessment was performed during a subsequent phase of data collection in the same cohort previously evaluated for depressive and anxiety symptoms, enabling an integrated analysis of fatigue severity, psychological symptoms, and disease activity within a single patient population. As all data were collected within the same clinical setting and during routine follow-up visits, the risk of selection and temporal bias was minimized. No additional data were collected specifically for this secondary analysis. Both original study protocols received approval from the Institutional Ethics Committee of the University Clinical Center Tuzla (approval number: 02-09/2-192-3/2025 and 02-09/2-193-3/2025) and were conducted in accordance with the Declaration of Helsinki.

Measures

Fatigue

Fatigue was assessed using the validated Functional Assessment of Chronic Illness Therapy-Fatigue (FACIT-F) questionnaire. This instrument was originally developed to assess fatigue in patients with cancer and anemia and was subsequently validated for use in patients with rheumatoid arthritis. The FACIT-F is a 13-item self-administered questionnaire that evaluates fatigue and its impact on physical, functional, emotional, and social well-being over the past seven days, with higher scores indicating lower fatigue severity. Each item is scored on a 0-4 Likert scale, with selected items reverse-scored, yielding a total score ranging from 0 to 52. Fatigue severity was categorized as none or minimal (>40), mild (>30 to ≤40), moderate (>21 to ≤30), and severe (≤21) [12]. The officially licensed Bosnian version of the FACIT-F was used with permission from FACIT.org. The instrument is copyrighted by David Cella, PhD. The primary outcome of this analysis was fatigue measured as a continuous FACIT-F score.

Depressive and Anxiety Symptoms

Depressive symptoms were assessed using the Beck Depression Inventory-II (BDI-II), and anxiety symptoms were assessed using the Beck Anxiety Inventory (BAI). The BDI-II comprises 21 items, each scored from 0 to 3, reflecting depressive symptoms over the previous two weeks. The total score ranges from 0 to 63, with higher scores indicating more severe depressive symptoms [13]. The BAI also consists of 21 items, each rated from 0 to 3 based on the extent to which the symptom bothered the individual during the past week. The total score ranges from 0 to 63, with higher scores indicating greater severity of anxiety symptoms [14]. For descriptive purposes, BDI-II and BAI scores were grouped according to standard cut-offs (BDI-II: 0-13 minimal, 14-19 mild, 20-28 moderate, and ≥29 severe; BAI: 0-7 minimal, 8-15 mild, 16-25 moderate, and ≥26 severe). For subsequent analyses, scores were treated as continuous variables.

Clinical and Demographic Variables

Disease activity was assessed using the Disease Activity Score in 28 joints (DAS28-ESR), a standardized and widely validated measure of disease activity in rheumatoid arthritis. It is calculated from four components: tender joint count, swollen joint count, the patient’s visual analogue scale (VAS) score for global health, and the erythrocyte sedimentation rate (ESR) [15,16]. Age and disease duration were recorded in years, and sex was recorded as a categorical variable. Disease duration was defined as the number of years since the diagnosis of rheumatoid arthritis.

Statistical analysis

All statistical analyses were conducted using R (R Foundation for Statistical Computing, Vienna, Austria). Continuous variables were summarized as medians and interquartile ranges (IQRs), while categorical variables were presented as frequencies and percentages. The Shapiro-Wilk test was used to assess the normality of continuous variables. As FACIT-F, BDI-II, and BAI scores were not normally distributed, nonparametric methods were applied. Associations between continuous variables were evaluated using Spearman’s rank correlation coefficient (ρ).

The primary objective of the regression analysis was to estimate independent associations with fatigue. Multivariable linear regression models were constructed with the FACIT-F score as the dependent variable. Disease activity (DAS28) was included as a covariate to account for inflammatory burden, while age, sex, and disease duration were included as clinically relevant variables.

Sequential models were specified to assess the incremental contribution of psychological variables: Model 1: FACIT-F ~ DAS28 + Age + Sex + Disease duration Model 2: FACIT-F ~ DAS28 + Age + Sex + Disease duration + BDI-II + BAI

This approach enabled the evaluation of the additional explanatory value of depressive and anxiety symptoms beyond biological factors. Model assumptions were assessed following model specification. Linearity and homoscedasticity were evaluated by visual inspection of residual plots. Multicollinearity was assessed using variance inflation factors (VIFs). VIF values ranged from 1.11 to 1.98, indicating no concerning multicollinearity among predictors included in the regression models. Robust heteroskedasticity-consistent (HC3) standard errors were applied to ensure conservative inference. Given the cross-sectional design, the analysis was not intended to establish causal relationships. Potential residual confounding by unmeasured variables, such as pain intensity and sleep disturbances, cannot be excluded.

No imputation procedures were required, as complete questionnaire and clinical data were available for all included participants. A formal sample size calculation was not performed, as the analysis was based on a predefined cohort from previously conducted studies. A p-value < 0.05 was considered statistically significant.

## Results

A total of 90 patients with rheumatoid arthritis were included in the analysis. Baseline characteristics of the study population are presented in Table 1. The median age of participants was 60 years (IQR: 49.3-64.8), and the median disease duration was 8.5 years (IQR: 3-17). The cohort was predominantly female, with 75 (83.3%) women.

**Table 1:** Baseline Characteristics of the Study Population (N = 90) Continuous variables are presented as median (IQR). Categorical variables are presented as number (percentage). IQR: interquartile range

Characteristic	Value
Age, years	60 (49.3-64.8)
Disease duration, years	8.5 (3-17)
Sex, number (%)	
Female	75 (83.3%)
Male	15 (16.7%)

The median DAS28 score was 4.62 (IQR: 3.82-5.37). The median FACIT-F score was 37.0 (IQR: 27.0-45.0). Higher FACIT-F scores indicate lower fatigue severity. Moderate-to-severe fatigue was observed in 32 (35.6%) patients. The median BDI-II score was 11 (IQR: 4.0-16.75), and the median BAI score was 13 (IQR: 5.25-18.75).

The distribution of fatigue, depressive, and anxiety symptom severity categories is presented in Table 2. Minimal fatigue was observed in 37 (41.1%) patients, mild fatigue in 21 (23.3%) patients, moderate fatigue in 23 (25.6%) patients, and severe fatigue in 9 (10.0%) patients. Moderate-to-severe depressive symptoms were present in 18 (20.0%) patients, whereas moderate-to-severe anxiety symptoms were observed in 33 (36.6%) patients.

**Table 2:** Distribution of Fatigue, Depressive, and Anxiety Symptom Severity in Patients With Rheumatoid Arthritis (N = 90) Severity categories were defined according to established cut-off values: FACIT-F (>40 minimal, >30-≤40 mild, >21-≤30 moderate, and ≤21 severe); BDI-II (0-13 minimal, 14-19 mild, 20-28 moderate, and ≥29 severe); BAI (0-7 minimal, 8-15 mild, 16-25 moderate, and ≥26 severe). FACIT-F: Functional Assessment of Chronic Illness Therapy-Fatigue, BDI-II: Beck Depression Inventory-II, BAI: Beck Anxiety Inventory

Severity level	FACIT-F, number (%)	BDI-II, number (%)	BAI, number (%)
Minimal	37 (41.1%)	54 (60.0%)	28 (31.1%)
Mild	21 (23.3%)	18 (20.0%)	29 (32.2%)
Moderate	23 (25.6%)	17 (18.9%)	21 (23.3%)
Severe	9 (10.0%)	1 (1.1%)	12 (13.3%)

The Shapiro-Wilk test indicated non-normal distribution for FACIT-F, BDI-II, BAI, age, and disease duration (p < 0.05), while DAS28 was normally distributed (p = 0.895). Spearman correlation analysis demonstrated a moderate negative correlation between FACIT-F scores and disease activity (DAS28) (ρ = -0.38, p < 0.001). Strong negative correlations were observed between FACIT-F scores and depressive symptoms (BDI-II) (ρ = -0.63, p < 0.001), as well as anxiety symptoms (BAI) (ρ = -0.60, p < 0.001). No significant correlations were identified between fatigue and age (ρ = 0.02, p = 0.83) or disease duration (ρ = 0.01, p = 0.93). Detailed results are presented in Table 3.

**Table 3:** Spearman Correlations Between Fatigue Severity (FACIT-F) and Clinical and Psychological Variables (N = 90) Spearman’s rank correlation coefficient (ρ) was used to assess associations between variables. FACIT-F: Functional Assessment of Chronic Illness Therapy-Fatigue (higher FACIT-F scores indicate lower fatigue severity), BDI-II: Beck Depression Inventory-II, BAI: Beck Anxiety Inventory, DAS28: Disease Activity Score in 28 joints

FACIT-F versus variable	Spearman ρ	p-value
FACIT-F versus BDI-II	-0.63	<0.001
FACIT-F versus BAI	-0.60	<0.001
FACIT-F versus DAS28	-0.38	<0.001
FACIT-F versus age	0.02	0.83
FACIT-F versus disease duration	0.01	0.93

To evaluate independent predictors of fatigue, two sequential multivariable linear regression models were constructed. In Model 1, higher disease activity (β = -2.75, p = 0.001) and female sex (β = -7.44, p = 0.003) were associated with lower FACIT-F scores, indicating greater fatigue. Age and disease duration were not significantly associated with fatigue (Table 4).

**Table 4:** Model 1: Biological Predictors of Fatigue Severity β represents the unstandardized regression coefficient indicating the change in FACIT-F score associated with a one-unit increase in the predictor. 95% CI: 95% confidence interval, FACIT-F: Functional Assessment of Chronic Illness Therapy-Fatigue (higher scores indicate lower fatigue severity); DAS28: Disease Activity Score in 28 joints Robust heteroskedasticity-consistent (HC3) standard errors were used. R² = 0.198; adjusted R² = 0.171

Predictor	β	95% CI	p-value
DAS28	-2.75	-4.44 to -1.06	0.001
Female sex (versus male)	-7.44	-12.26 to -2.62	0.003
Age	-0.04	-0.22 to 0.14	0.650
Disease duration	0.06	-0.21 to 0.33	0.667

Model 2 extended the biological model by including depressive and anxiety symptoms (BDI-II and BAI). After inclusion of psychological variables, both depressive symptoms (β = -0.48, p = 0.008) and anxiety symptoms (β = -0.40, p = 0.002) remained independently associated with fatigue. In this model, the association between DAS28 and fatigue was attenuated and was no longer statistically significant. The association with female sex was also no longer statistically significant (Table 5).

**Table 5:** Model 2: Biological and Psychological Predictors of Fatigue Severity β represents the unstandardized regression coefficient. 95% CI: 95% confidence interval, BDI-II: Beck Depression Inventory-II, BAI: Beck Anxiety Inventory, FACIT-F: Functional Assessment of Chronic Illness Therapy-Fatigue Robust heteroskedasticity-consistent (HC3) standard errors were applied. R² = 0.524; adjusted R² = 0.495

Predictor	β	95% CI	p-value
DAS28	-1.27	-2.66 to 0.12	0.072
Female sex (versus male)	-1.62	-5.40 to 2.16	0.400
Age	0.03	-0.11 to 0.17	0.677
Disease duration	0.06	-0.14 to 0.26	0.553
BDI-II	-0.50	-0.87 to -0.13	0.008
BAI	-0.40	-0.66 to -0.15	0.002

Model comparison demonstrated that the inclusion of psychological variables substantially improved model fit. Model 1 explained 19.8% of the variance in FACIT-F scores (adjusted R² = 0.171), whereas Model 2 explained 52.4% of the variance (adjusted R² = 0.495). Depressive and anxiety symptoms accounted for an additional 32.6% of the variance in fatigue (ΔR² = 0.326), as shown in Table 6.

**Table 6:** Incremental Explained Variance in Fatigue Severity ΔR² represents the increase in explained variance in FACIT-F scores after the inclusion of psychological variables (BDI-II and BAI) in Model 2 compared with Model 1. FACIT-F: Functional Assessment of Chronic Illness Therapy-Fatigue, BDI-II: Beck Depression Inventory-II, BAI: Beck Anxiety Inventory

Model	R²	Adjusted R²	ΔR²
Model 1 (Biological)	0.198	0.171	-
Model 2 (Full)	0.524	0.495	0.326

## Discussion

The present study demonstrated that fatigue in rheumatoid arthritis (RA) is associated with depressive and anxiety symptoms and that these associations persist independently of inflammatory disease activity. Although disease activity showed a moderate association with fatigue severity, psychological variables remained independently associated with fatigue severity after multivariable adjustment. An additional strength of the present study is the simultaneous evaluation of biological and psychological variables within the same patient cohort using sequential multivariable models, enabling assessment of their relative contributions to fatigue severity.

Consistent with previous literature, higher disease activity was associated with greater fatigue severity [1,2,4,17]. However, this association was attenuated and lost statistical significance after adjustment for depressive and anxiety symptoms, suggesting that the association between inflammatory activity and fatigue may be influenced by psychological factors. Similarly, the association between female sex and fatigue was no longer statistically significant after adjustment for psychological variables. This attenuation may suggest partial mediation through depressive and anxiety symptoms, although this interpretation should be considered exploratory given the cross-sectional design. Similar findings have been reported in previous studies, in which fatigue was only partly explained by biological disease activity and appeared to be more closely related to pain and psychological distress [4,17-19].

A key finding of this study was the marked increase in explained variance after the inclusion of depressive and anxiety symptoms in the regression model (ΔR² = 0.326). Psychological variables increased the model’s overall explanatory capacity from 19.8% to 52.4%, indicating that depressive and anxiety symptoms account for a substantial proportion of fatigue severity beyond inflammatory and demographic factors. These findings are consistent with previous studies identifying psychological distress as an important contributor to fatigue in RA [5-8,20].

The present findings can be interpreted within the biopsychosocial framework of fatigue, in which biological, psychological, and behavioral factors interact dynamically [11,20]. Contemporary network-based models further suggest that symptoms such as fatigue, pain, depression, anxiety, and sleep disturbances do not occur independently, but rather influence one another through reciprocal interactions [11,21]. Within this framework, psychological distress may amplify the severity of fatigue, while persistent fatigue may contribute to ongoing psychological distress, potentially contributing to a persistent symptom burden. Several biological mechanisms proposed in previous literature may contribute to the observed associations between psychological symptoms and fatigue. Pro-inflammatory cytokines, including interleukin-6 and tumor necrosis factor, may influence central nervous system signaling, neuroendocrine regulation, and neurotransmitter pathways involved in mood and fatigue perception [9,22-24]. Alterations in hypothalamic-pituitary-adrenal axis activity and serotonergic or dopaminergic signaling have also been implicated in the development of central fatigue and depressive symptoms in chronic inflammatory diseases [22-24].

From a clinical perspective, these findings suggest the limitations of a purely inflammation-centered approach to fatigue assessment and management in RA. Pharmacologic control of inflammatory disease activity alone may be insufficient to substantially reduce fatigue in a proportion of patients [17-19]. Current EULAR recommendations emphasize multimodal management strategies that integrate psychological support, physical activity, patient education, and optimization of overall functional health [25]. Consideration of depressive and anxiety symptoms may therefore be relevant in patients reporting persistent fatigue despite treatment of inflammatory disease activity.

Moderate-to-severe fatigue was present in 32 (35.6%) patients in this cohort, which was broadly consistent with previous reports, although somewhat lower than the highest reported prevalence estimates [1,2,18]. Nevertheless, fatigue remains a major unmet clinical need in RA because of its substantial impact on quality of life, daily functioning, and overall patient well-being.

This study has several limitations. First, the cross-sectional design precludes causal inference, and the directionality of the observed associations cannot be determined. Second, the study was conducted at a single center with a relatively modest sample size and a predominance of female participants, which may limit the generalizability of the findings. Variability in disease activity within the cohort may also have contributed to heterogeneity of the results. In addition, no healthy control group or disease-control cohort was included, limiting direct comparisons of fatigue and psychological symptom burden with the general population or other chronic disease populations. All measures were based on patient-reported questionnaires, and fatigue, depression, and anxiety are inherently subjective experiences that may be influenced by reporting bias and individual symptom perception, despite the use of validated standardized instruments. Residual confounding by unmeasured variables, including pain intensity, sleep disturbances, physical activity, and comorbid fibromyalgia, cannot be excluded. In addition, the relatively modest sample size may have limited the statistical power to detect smaller associations and interaction effects. Although all assessments were performed within the same clinical setting and follow-up period, the possibility of temporal variability between the earlier psychological assessments and subsequent fatigue assessment cannot be fully excluded.

## Conclusions

Fatigue in rheumatoid arthritis appears to be associated not only with inflammatory disease activity but also with depressive and anxiety symptoms. These findings support a multidimensional biopsychosocial understanding of fatigue and indicate that psychological factors contribute importantly to fatigue severity in rheumatoid arthritis. Consideration of psychological symptoms may be relevant when evaluating persistent fatigue in patients with rheumatoid arthritis despite treatment of inflammatory disease activity.
